# Verifying participant-reported clinical outcomes: challenges and implications

**DOI:** 10.1186/s13063-020-4169-7

**Published:** 2020-03-04

**Authors:** Suzanne Breeman, Lynda Constable, Anne Duncan, Kath Starr, Alison McDonald, Samantha Wileman, Seonaidh Cotton

**Affiliations:** Centre for Healthcare Randomised Trials (CHaRT), Health Services Research Unit (HSRU), University of Aberdeen, Health Sciences Building, Foresterhill, Aberdeen, AB25 2ZD Scotland

**Keywords:** RCT, Verification, Patient reported clinical outcomes, Accuracy

## Abstract

**Background:**

Researchers often rely on trial participants to self-report clinical outcomes (for example, fractures, re-operations). Little information exists as to the ‘accuracy’ of participant-reported clinical outcomes, particularly in randomised controlled trials (RCTs). To help address this evidence gap, we report four case studies, nested within different RCTs where participant-reported clinical outcome data were compared with those reported by clinicians or extracted from medical notes.

**Methods:**

Four publicly-funded RCTs with different methods of verifying participant-reported outcomes were identified. In KAT, the participants were asked about hospital admissions for any reason. Where it was thought to be relevant to the trial knee, further information was sought from the lead surgeon at the admitting site to confirm whether or not the admission was relevant to the trial knee. In REFLUX, participants were asked about hospital admissions for any reason. For participants who reported a re-operation, further information was sought from the lead surgeon at the admitting site to confirm this. In RECORD, participants were asked three questions regarding broken bones. Where low-trauma fractures were reported, clinical verification was sought, initially from the research nurse at the site.

In CATHETER, participants were asked about urinary tract infections (UTIs), and a prescription of antibiotics was provided for the treatment of UTIs following urethral catheterisation. The GPs of those who reported a UTI were contacted to confirm that an antibiotic prescription had been issued for the suspected UTI.

**Results:**

In KAT, 397 of 6882 (6%) participant-reported hospital admissions were confirmed as relevant to the trial knee. In REFLUX, 16 of 19 participants (84%) who appeared to have had a re-operation were confirmed as having had one. In RECORD, 473 of 781 (61%) fractures reported by participants were confirmed as being low-trauma fractures. In CATHETER, 429 of 830 participant-reported UTIs (52%) were confirmed as such by the GPs.

**Conclusions:**

We used different approaches in our verification of participant-reported outcomes in clinical trials, and we believe there is no one optimal solution. Consideration of issues such as what information is sought from participants, the phrasing of questions, whether the medical records are a true ‘gold standard’ and costs and benefits to the RCT may help determine the appropriate approach.

## Background

In both research and in routine health service provision, a review of outcomes following medical intervention (including, for example, drug treatment, surgery, psychological interventions) is important to assess the effects of the intervention. These outcomes may include quality of life, disease-specific symptoms, impairment, disability, results of laboratory tests, complications of treatment and the need for further treatment [1–3].

Within a research context, self-reporting of clinical outcomes (for example fracture, re-operation, and hospitalisation) by participants is common (for example Costa et al. [4], Ashby et al. [5]). Many reasons may explain this, including cost and convenience for the researchers or that participant-reported data are becoming more widely accepted. However, little information exists on the ‘accuracy’ of participant-reported clinical outcomes [6], particularly in the context of clinical trials. Existing research, nested in observational or registry-based studies, has tended to compare participant report against reports made by medical professionals or otherwise captured in the medical records [3, 7–9].

Previous studies have shown variable concordance between participant report and clinical report. A review comparing the accuracy of questionnaire data compared to medical records in observational studies suggest the fairly accurate recall of events such as hospitalisation and surgery but poorer recall of, for instance, diagnostic X-ray [7]. In a registry study comparing participant and surgeon-reported complications following hip arthroplasty, concordance varied across different complications; for example, for pulmonary embolism, concordance was 89% compared to 32% for bleeding [8]. Similar rates were reported by Dushey et al. (pulmonary embolism 84%; bleeding 37%) in a registry-based study that compared participant- and surgeon-reported complications following knee replacement [3]. Alazzawi et al. reported approximately 80% accuracy of participant-reported complications in a single centre study of total hip and knee replacement. They also noted that some types of post-discharge complications appear to be more accurately reported by participants than others. For example, the need for further surgery was accurately reported by 100% of respondents, compared to 75% for myocardial infarction, and 36% for nerve injury [9].

Following adjudication of cardiovascular events reported by participants or a family member in a large randomised controlled trial (RCT) of calcium supplementation, 58% (50 of 86) of self-reported strokes and 52% (33 of 64) of self-reported myocardial infarctions were verified from the clinical records [6].

To help address the evidence gap in relation to the accuracy of participant-reported clinical outcomes within the context of pragmatic RCTs, we report four case studies, nested within different RCTs (termed the parent trials and summarised in Table 1), where participant-reported clinical outcome data were compared with those reported by clinicians or in medical notes. Within each case study, we present the participant-reported clinical outcomes collected, details of the verification process and the results of this process. Each of the verification processes were undertaken as part of the data collection component of the parent trial. We then discuss some common themes emerging from these observations.

**Table 1:** Summary of parent trials

Trial	Design and comparisons	Recruitment
The Knee Arthroplasty Trial (KAT) [10] ISRCTN45837371 NIHR Health Technology Assessment Programme	Partial factorial, pragmatic, multicentre RCT comparing three aspects of knee replacements: resurfacing the patella vs no resurfacing; mobile bearing between the tibial and femoral components vs standard designs without a mobile bearing; and metal-backed plate for the tibial component vs single high-density- polyethylene component.	July 1999 to January 2003, 2352 participants randomised: Resurfacing patella (*n* = 1715) Mobile bearing (*n* = 539) Metal backing (*n* = 409) 345 participants randomised in more than one comparison.
The REFLUX trial [11] ISRCTN15517081 NIHR Health Technology Assessment Programme	Pragmatic, multi-centre RCT to evaluate the clinical effectiveness, cost effectiveness and safety of a policy of relatively early laparoscopic surgery compared with continued, but optimised medical management amongst people with gastro-oesophageal reflux disease (with contemporaneous patient preference arms).	March 2001 to June 2004, 357 participants randomised: Surgery (*n* = 178) Medical management (*n* = 179). 453 participants entered the preference arm: Preferred surgery (*n* = 261) Preferred medical management (*n* = 192)
The RECORD trial [12] ISRCTN51647438 UK Medical Research Council	Factorial, pragmatic, multi-centre RCT to assess whether 800 IU daily oral vitamin D3 and 1000 mg calcium, either alone or in combination, were effective in prevention of secondary fractures.	January 1999 to March 2002, 5292 participants randomised: Vitamin D and calcium (*n* = 1306) Vitamin D alone (*n* = 1343) Calcium alone (*n* = 1311) Placebo (*n* = 1332)
The CATHETER trial [13] ISRCTN75198618 NIHR Health Technology Assessment Programme	Pragmatic, multi-centre randomised controlled trial to evaluate the clinical and cost effectiveness of three different types of urethral catheters (antiseptic-coated (silver) latex catheter, antimicrobial-impregnated (nitrofurazone) catheter or a standard PTFE-coated latex catheter) to reduce catheter-associated urinary tract infections (UTIs).	July 2007 to September 2010, 6394 participants randomised and included: Silver alloy catheter (*n* = 2097) Nitrofurazone catheter (*n* = 2153) PTFE catheter (*n* = 2144)

## Case study 1 - The Knee Arthroplasty Trial (KAT) [10]

### Clinical outcome data and verification

Disease-specific and general quality of life, GP prescribing data, physiotherapy and outpatient consultations related to the trial knee and hospital admissions (for any reason) were collected by postal questionnaires completed by participants at baseline, 3 months after surgery, 1 year after and annually thereafter to 20 years (the 13-year follow-up was complete for all participants at the time of data extraction). Participants were asked to provide a brief description of all hospital readmissions (although the study was only interested in those relevant to the trial knee). All participant-reported hospital readmission information was uploaded onto the trial database.

The KAT nurse coordinator reviewed all the hospital readmissions to identify those that were potentially related to the participant’s trial knee (participants admitted for bilateral knee replacements nominated the most painful knee as the one to be considered for randomisation) and for these re-admissions, wrote to the lead surgeon/hospital where the readmission occurred and asked for further details. A proforma was designed and completed by the site to capture important information (for example, was the participant readmitted, did the participant require further knee surgery) which allowed the readmissions to be classified as either ‘relevant’ or ‘not relevant’ to the trial knee.

### Results of the verification

Of all hospital readmissions recorded by participants in their questionnaires, only 6% (397/6882) were confirmed as relevant (i.e., related to the trial knee). Most readmissions (94%; 6436/6882) were either deemed to be not relevant (i.e., not related to the trial knee) by the nurse coordinator and therefore not followed up or confirmed as not relevant by the lead surgeon/hospital where the readmission took place. In 0.7% (49/6882) of the cases, verification from the lead surgeon/hospital was ongoing at the time of writing.

## Case study 2: REFLUX [11]

### Clinical outcome data and verification

Participants were followed up with annual postal questionnaires for a minimum of 5 years. The questionnaire collected information about disease-specific and generic quality of life; reflux medication use; and any GP visits, hospital outpatient consultations, or day and overnight hospital admissions in the previous year; and the reasons for these (including repeat surgery).

One of the key outcomes of the trial was reflux-related re-operation for reflux symptoms; differentiating between true cases of repeat surgery and other unrelated surgical procedures was considered important. Four types of re-operations were considered: conversion (to a different type of wrap), reconstruction of the same wrap, reversal of the fundoplication or repair of hiatus hernia only. From the free text provided by participants in their questionnaires, confirmation that surgery re-operation had taken place or of the type of re-operation was not always possible. From the self-reported data, we identified participants who appeared to have had repeat surgery, and the Chief Investigator and Lead Surgeon wrote to the surgeon at the site to ask for further details about the surgery. We designed a proforma to capture the relevant information, which included (i) whether any record existed of a re-operation, (ii) what type of hernia was involved, (iii) what type of re-operation was undertaken, and (iv) the reasons for the re-operation. If there was no response to the initial request, a reminder letter was sent.

### Results of the verification

From the participant self-report, we identified 19 participants who appeared to have had a re-operation. Three of the participants were confirmed as not having had repeat surgery. The remaining 16 were confirmed as having had repeat surgery, and we were able to classify the type of repeat surgery (for example reconstruction, conversion etc). We also confirmed that two of the participants had a third re-operation.

## Case study 3: The RECORD trial [12]

### Clinical outcome data and verification

Following randomisation, participants were asked to complete postal questionnaires every 4 months until all participants had been followed up for a minimum of 24 months. The questionnaires contained three questions that capture the primary outcome for the trial - new low-trauma fractures including clinical, radiologically confirmed, vertebral fractures (excluding those of the face and skull). Fractures reported by participants were verified by research nurses at the sites where possible. Furthermore, to try to ensure as complete a dataset as possible (due to participant dropout/non-response to postal questionnaires/admission occurring at a non-RECORD hospital), a survey was conducted of GPs of all trial participants at the end of follow-up to ask about further fractures. Hospital episode statistics (HES) and Information Services Division (ISD) data were also used in the verification process.

### Results of the verification

Participants reported 781 broken bones, of which 489 were deemed potentially relevant by the trial team and were subject to verification. The other fractures that were reported by participants were not considered relevant to the primary outcome and therefore were not further verified. Four hundred and forty-five of the 489 fractures were subsequently verified from X-ray reports in primary and secondary care records, 28 were verified from clinical diagnosis recorded in primary and secondary care records, and the 16 remaining fractures reported could not be verified from primary or secondary care sources.

## Case study 4 – CATHETER [13]

### Clinical outcome data and verification

The primary outcome for the RCT was the incidence of catheter-associated urinary tract infection (CAUTI) in the 6 weeks following randomisation. This was defined as participant-reported symptoms of urinary tract infection (UTI) with a prescription of antibiotic for a UTI. Data were collected via postal questionnaires and diaries at 3 days, 1 week and 2 weeks post-catheter removal and 6 weeks post-randomisation. During the trial, the DMC noted a higher than expected proportion of participants reporting the primary outcome. Further investigation revealed that this may have been due to uncertainty on the part of the participant about their prescription and the purpose of the antibiotics they received (e.g., prophylaxis post-surgery rather than to treat a suspected UTI). Therefore, the GPs of participants who reported a UTI were contacted to request confirmation that an antibiotic prescription had been issued and that this was for a suspected UTI.

GPs were asked to complete a proforma with information on whether the participant had been diagnosed with a UTI during the time they were in the trial, and whether they had received a prescription for antibiotics for a UTI during that time. The proforma was sent to a named GP, with a reminder if no response was received. Further telephone reminder calls were made from the trial office to GPs who failed to respond to the reminder letter. In addition, the Scottish Primary Care Research Network and Primary Care Research Network in England (SPCRN and PCRN, respectively) nurses were contacted to help elicit a response or collect the required information from non-responding GP practices.

### Results of the verification

The GPs of 830 participants were contacted to verify participant-reported UTIs. Of the GPs contacted to confirm participant-reported UTI and antibiotic prescription, 429 of 830 (51.7%) confirmed the participant information was correct, and 393 (47.3%) confirmed there had been no UTI. Overall, the presence/absence of UTI could not be confirmed by the GP or the GP did not agree to provide the information for eight participants (1.0%).

## Discussion

In this paper we have demonstrated different approaches to the clinical verification of participant-reported outcomes that have been used with varying success in our clinical trials unit. We have developed the approaches to verification on a trial-by-trial basis to accommodate the requirements of the individual trial, the type of participant-reported clinical outcome and the available resources.

The outcomes of interest have been varied and include related hospitalisations, repeat surgery, further low-trauma fractures and post-catheterisation UTI. Because of the diverse nature of the trials in which our case studies were nested and the different approaches to verification used, we did not set out to assess whether some clinical outcomes were more accurately reported by participants than others. However, limited data from other studies suggest that this may be the case [6–9]. A possible reason for this may be that participants find some outcomes easier to report than others because of the terminology and language used in the questionnaire, how familiar the participants are with the outcome or what they are told by medical staff. For example, in the CATHETER case study, some participants were given prophylactic antibiotics and may have thought this was to *treat* rather than to *prevent* an infection. We are aware of one previous study comparing participant and clinician reports of UTI, which showed 82 and 84% agreement at 3 and 12 months post-prostatectomy [14], but it is not clear what question(s) participants were asked in order to compare this data or what the clinical definition of a UTI was. In CATHETER, participants were asked ‘have you had a urine infection’ and ‘have you received antibiotics for a urine infection’. The trial, therefore, likely identified symptomatic UTIs where participants sought treatment and prophylactic antibiotics rather than asymptomatic microbiologically confirmed UTIs.

A further mismatch may have occurred in the participant’s and clinical/research staff’s understanding (or misunderstanding) of a medical term. For example, in CATHETER, participants were asked which antibiotic they had been prescribed, and in a number of cases, the participant could not recall the name, or described a drug that was either not an antibiotic or would not be used to treat UTIs. An alternative approach may have been to list the antibiotics used to treat UTIs and ask the participants whether they had received any of these. Alazzawi provide an example of four participants incorrectly reporting that they had a stroke when they had experienced a transient ischaemic attack (TIA) [9]. In a population-based survey, only 29% of those reporting a stroke had this verified by hospital records [15]. Participants who experience a TIA may understand that they have had ‘mini-stroke’, but not recognise that this is the same as a TIA, so such misunderstandings are perhaps unsurprising. Furthermore, participants may not be provided with sufficient information about a medical term to accurately record the outcome of interest. For example, Dushey et al. [3] noted 36.7% accuracy of participant-reported major bleed episodes: the clinical verification undertaken followed strict criteria, but participants were not given any criteria as to what constituted a major bleed.

The timing by which participants are asked to report an outcome (in relation to both the outcome itself and in relation to the original event) may also impact on the accuracy of their reporting. Other factors that may impact on the accuracy of participant-reported clinical outcomes is how relevant the outcome is to the participant themselves or how much impact it has on their life. The need for repeat surgery is likely to have a bigger impact on the participant’s life than, for example, a urinary tract infection.

Within this piece of work, we set out to verify the clinical events that were reported by participants. Participants may, of course, have experienced relevant clinical events that they did not report in a questionnaire. When relying on participant-reported outcomes only, the magnitude of this is not known. This may be a limitation of the verification methodology more generally.

A further limitation (of our case studies, and previous work in this area) is that the ‘clinical verification’ provided by medical records may not be a true gold standard. For example, the records themselves may be subject to inaccuracies.

An alternative to verifying participant-reported outcomes using individual patient’s medical records would be to confirm them using routine datasets, but again limitations may exist in such an approach. Routine data may be subject to inaccuracies; for example, Information Services Division (ISD) has a standard for accuracy of routine data of 90%, and for general/acute inpatient and day cases (SMR01) they report and accuracy of 89–94% [16]. Possible reasons for inaccuracy of routine data include operations being miscoded (perhaps due to a lack of clinical engagement in coding [17]) or operations being coded to the wrong participant. For certain conditions, other limitations may exist in relation to using routine data to verify participant-reported clinical outcomes. First, insufficient information may exist to enable one to identify which limb was involved or to identify the specific types of operation. Second, operations carried out privately will not be captured in routine data, which may be particularly problematic if this constitutes a large proportion of the operations. A third consideration relates to potential time-lags in routine data being coded and made available to researchers, and in the approvals process to access routine data for research purposes. Fourth, participants may not have given consent for use of routine data at the outset of the trial. Finally, some outcomes are not routinely captured in national datasets. For example, for the CATHETER outcome (UTI), no national register exists, and although general national prescribing data could be used to provide information about antibiotic prescriptions, this information could not be linked to individuals. Although routine data were available for KAT and data about all participants were requested from GPs for RECORD, in this paper, we did not attempt to validate these sources.

Considering that inaccuracies may exist in both participant-reported clinical outcomes and in data captured in medical records, decisions have to be made in terms of which source to treat as ‘correct’. In the four case studies presented here, different approaches were taken. In KAT [10] and REFLUX [11], the information from the clinician (GP or surgeon) was considered to be the ‘correct’ information, and unless the event was verified by the GP or surgeon, it was not included in the analysis. In RECORD [12], clinical verification of fractures reported by participants was sought (from the recruiting site, GP and/or central data sources). Fractures that could not be confirmed (*n* = 16) were not included in the main analysis. In CATHETER [13], where GP information was received, it was regarded as the definitive data. However, in the few (eight of 830 participants) of participant-reported UTIs that could not be confirmed by the GP (for example, no response was received from the GP, or the participant was no longer registered with the GP practice), the researchers used alternative sources (i.e., responses to other questions within the questionnaire) to verify the data. Therefore, before a process to verify participant-reported clinical outcomes is undertaken, strategies to deal with mismatches in data should be considered and documented.

The potential benefit of verification of participant-reported clinical outcomes is difficult to quantify, and again, such quantification is not something we set out to do in this paper. Advantages may exist in terms of the external generalisability of the results to the population, which can make the results appear more relevant to clinicians practicing in that area. Equally, there may be merit if there are particular safety concerns where accurate reporting is essential. However, less impact on the comparison of interventions is possible, particularly in studies where the participant is blinded to the intervention and therefore less likely to be biased by knowledge of their treatment allocation. Thus, greater benefit may exist in clinical verification of participant-reported outcomes in studies that are not blinded (where participants may have preconceptions about the intervention they have had). Similarly, if the outcome (and particularly any misclassification of outcome) is not equally distributed between arms of the parent trial, the benefit of verification may be greater.

Whilst the potential benefit may be hard to quantify, the costs involved in any approach to verification should be carefully considered. Of the 6882 clinical outcomes reported in participant questionnaires (KAT Trial), only approximately 6% were confirmed as being relevant (i.e., related to the trial knee) and included in the statistical analysis [10]. In RECORD, where they used three questions to capture the primary outcome (‘broken any bones’, ‘how did you break’ and ‘which bone(s)’) the trial team were better able to identify which reports were potentially relevant and should be subject to further verification [12]. Therefore, refining the question asked of KAT participants, i.e., asking the participants to report knee-related hospital readmissions only or ask a series of questions to capture additional/relevant information, may increase the proportion of events confirmed as relevant and reduce the time involved in coding and/or verifying those later identified as not relevant. Checking single cases against hospital or primary care records may simply involve a time-cost for the trial team and the clinical staff, which will be variable contingent on the number of cases being checked. For example, in REFLUX [11], only 19 surgeons were contacted for further information, compared to CATHETER [13], where the GPs of more than 800 participants had to be contacted. To facilitate the workload in CATHETER, a full-time member of staff was employed whose main duty to manage the process of collecting and processing the resulting data over a 3- to 6-month period. Both time and financial costs are associated with obtaining routine data and linking this to trial cohorts.

## Recommendations for practice

Our primary recommendation is that, during the planning phase of a trial, careful consideration be given not only to which participant-reported clinical outcomes should be collected and how these will be collected but also to whether any verification of these should be undertaken. The decisions reached are very likely to vary on a trial-by-trial basis; it is our opinion that there is no optimal, ‘one size fits all’ solution. However, making decisions about any clinical verification of participant-reported outcomes at the outset of the study will help ensure that any costs associated with verification are covered, that the trial time-line includes adequate time for collection and verification of outcomes, and that the consent sought from participants to allow any verification is sufficient. Furthermore, we recommend that consideration be given to how any discrepancies in outcome identified during the verification process will be addressed (for example whether unverified outcomes will be included in the analysis or not).

If trial participants are being asked to report clinical outcomes as part of a questionnaire, the use of appropriate language is important, so the participants can accurately respond about the clinical information that is being requested from them. The use of very ‘medical’ terms may be inappropriate. Careful piloting of such questions may help avoid misinterpretation, confusion or inaccurate responses.

## Recommendations for future research

Further reports on the approaches to and the impacts of clinical verification of participant-reported outcomes will help inform researchers. More formal cost-benefit type analyses, considering both the impact and the cost of verification, would further inform decisions about the relative value of verification in different settings.

## Conclusion

We have described a number of approaches to the clinical verification of participant-reported outcomes, and some of the issues that trialists may wish to consider when deciding whether such verification is necessary in a specific trial, and if so, what approach to this may be appropriate. We believe that there is no one optimal solution but that consideration of the issues presented here will help inform decisions made in future trials.

## Data Availability

The datasets used and/or analysed during the current study are available from the corresponding author on reasonable request.
